# Anti-Muscle-Specific Kinase (MuSK) Antibody-Positive Myasthenia Gravis Presenting With Dyspnea in an Elderly Woman: A Case Report

**DOI:** 10.7759/cureus.50480

**Published:** 2023-12-13

**Authors:** Tadaharu Shiozumi, Nobunaga Okada, Tasuku Matsuyama, Yoshihiro Yamahata, Bon Ohta

**Affiliations:** 1 Department of Emergency Medicine, Kyoto Prefectural University of Medicine, Kyoto, JPN; 2 Department of Emergency Medicine, Japanese Red Cross Society Kyoto Daiichi Hospital, Kyoto, JPN

**Keywords:** atypical presentation, elderly onset, musk antibody, respiratory failure, myasthenia gravis

## Abstract

Myasthenia gravis (MG) is an autoimmune disease and represents one of the most common disorders associated with neuromuscular transmission defects. Within MG, the anti-muscle-specific kinase antibody-positive subtype (MuSK-positive MG) is rare. While it shares similarities with the common form of MG by presenting with ocular weakness, MuSK-positive MG typically presents with more atypical symptoms. Although MuSK-positive MG can lead to type 2 respiratory failure due to respiratory weakness, there have been limited reports where initial presentation involves only respiratory compromise. This study details a case of MuSK-positive MG presenting dyspnea. An 84-year-old female presented to the emergency department due to a three-day history of progressive respiratory distress, characterized by increased respiratory effort and shallow breathing, resulting in a diagnosis of type 2 respiratory failure. Despite the absence of neurological abnormalities, she tested positive for anti-muscle-specific kinase antibodies, confirming a diagnosis of MuSK-positive MG. This case highlights the significance of considering MG in the context of type 2 respiratory failure, even in the absence of typical neurological symptoms, especially in elderly patients.

## Introduction

Myasthenia gravis (MG) is an autoimmune disease that results in impaired signal transmission due to the presence of pathogenic autoantibodies to several target antigens on the postsynaptic membrane of the neuromuscular junction. Antibodies against acetylcholine receptor (AChR) and muscle-specific kinase (MuSK) induce myasthenic weakness by inhibiting normal neuromuscular signaling. Although symptoms and response to treatment vary depending on the age of onset and antibodies involved, it typically begins with ocular symptoms such as ptosis and diplopia [1]. Within MG, the anti-muscle-specific kinase antibody-positive subtype (MuSK-positive MG) is rare, accounting for 5-8% of all cases [2]. Unlike AChR-MG, which often affects females in their 20s and males aged 60 or older, MuSK-positive MG often affects younger individuals, particularly females under 40 years, and rarely presents after the age of 70 years [3]. In addition, MuSK-MG is clearly predominant in females (78-100%) [4].

There are subtypes of MG defined by age of onset, including late-onset (50-64 years) MG and elderly-onset (≥65 years) MG, whose prevalence has increased recently [5]. A nationwide survey in Japan revealed a 1.5-fold increase in the incidence of late-onset MG over 19 years, with a 2.3-fold increase among elderly patients [6]. MG may be underdiagnosed in the elderly population, or the incidence of late-onset MG may be increasing, or possibly both [5]. Elderly-onset MG is more likely to be severe with life-threatening events at its onset. However, appropriate diagnosis and treatment lead to a good prognosis even in the elderly, as many factors, including comorbidities, medications, and age-related changes, can delay diagnosis [7]. In this report, we present a case of an elderly patient with MuSK-positive MG, who presented with isolated type 2 respiratory failure.

## Case presentation

An 84-year-old female presented to the emergency department with a three-day history of worsening respiratory distress. Her medical problems included reflux esophagitis, insomnia, and Alzheimer's disease. She had no history of obstructive lung pathology and no history of tobacco use. Her home medications consisted of lansoprazole, mosapride citrate hydrate, donepezil hydrochloride, memantine hydrochloride, brotizolam, and albumin tannate. There was no documented history of autoimmune diseases or significant family medical history related to these conditions. Additionally, no obvious triggers, such as recent infections or heightened stress levels, were identified.

**Figure 1:**
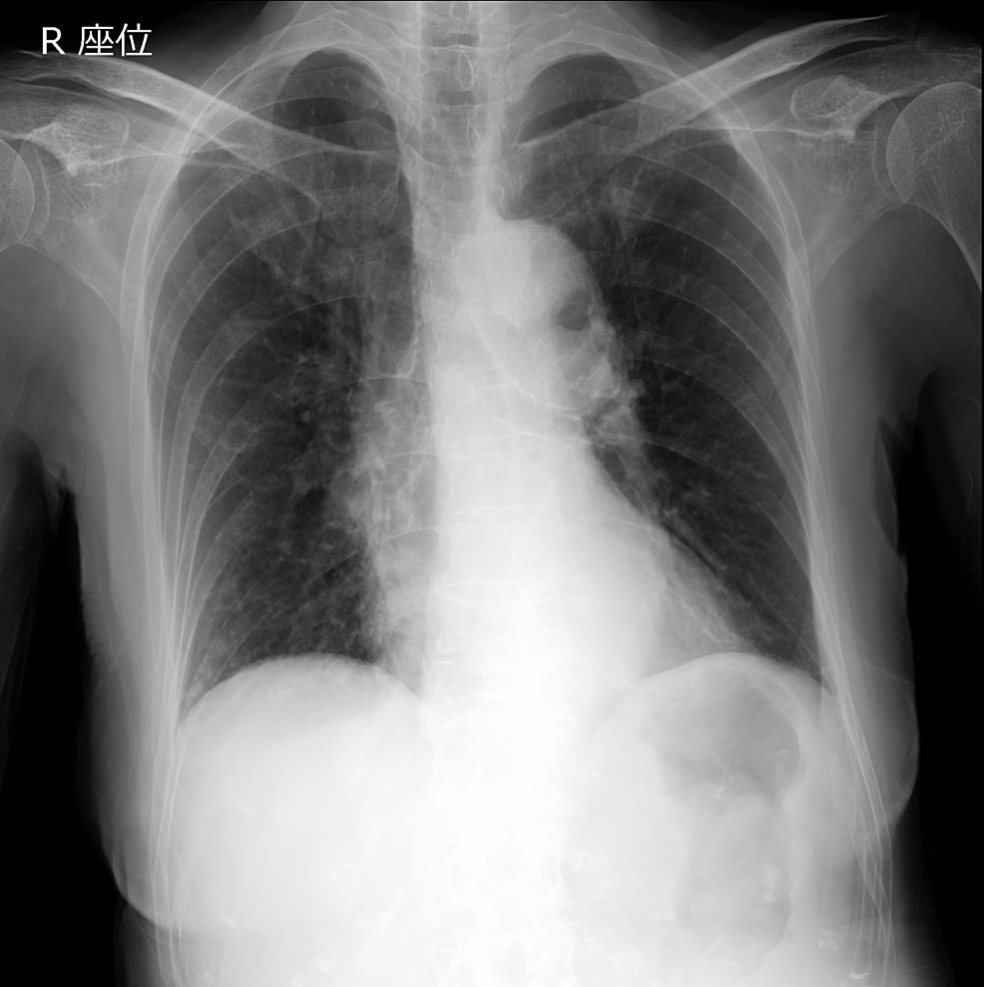
Chest x-ray showed no remarkable findings.

On initial evaluation in the emergency department, her vital signs were as follows: respiratory rate was 25 breaths/min, SpO_2_ 90% (on room air), blood pressure 189/109 mmHg, pulse rate 109 beats/min, and her body temperature was 36.5°C. Her Glasgow Coma Scale score was 15 (E4V5M6). Chest, cardiovascular, and abdominal examinations were unremarkable. No obvious neurological abnormalities could be noted. Arterial blood gas analysis on room air showed pH of 7.35, PaCO_2_ 64.9 mmHg, PO_2_ 51.0 mmHg, and HCO_3_- 35.4 mmol/L. Blood test showed hypokalemia (serum potassium level: 2.9 mEq/L); however, it was unclear whether this was related to her respiratory distress. The other laboratory evaluation showed no remarkable findings (Table 1). Her chest radiography findings were unremarkable (Figure 1).

**Table 1:** Results of blood cell count, biochemistry, arterial blood gas analysis, and autoantibody tests.

Blood test	Result	Reference range
White blood cells	9.4	3.3-8.6×10^3^/μL
Hemoglobin	13.7	11.6-14.8 g/dL
Platelet count	131	158-348×10^3^/μL
Sodium	139	138-145 mmol/L
Potassium	2.9	3.6-4.8 mmol/L
Chloride	96	101-108 mmol/L
Calcium	9.1	8.8-10.1 mg/dL
Blood urea nitrogen	11.4	8.0-20.0 mg/dL
Creatinine	0.39	0.46-0.79 mg/dL
Aspartate transaminase	38	13-30 U/L
Alanine transaminase	36	7-23 U/L
Brain natriuretic peptide	26.2	0.0-18.4 pg/mL
Arterial blood gas		
pH	7.345	7.35-7.45
PaCO_2_	64.9	32-45 mmHg
PaO_2_	51	83-108 mmHg
HCO_3_	35.4	22.0-26.0 mmol/L
Lactate	2.3	0.5-1.6 mmol/L
Antibody test		
Acetylcholine receptor antibodies	Not detected	0.0-0.2 nmol/L
Muscle-specific tyrosine kinase antibody	14.3	0.0-0.01 nmol/L
Anti-nuclear antibody	<40 (negative)	Negative: <40
Anti-double-stranded DNA antibody	<10 (negative)	Negative: <12.0 IU/mL
Sjogren's anti-SS-A	21.2	0.0-9.9 U/mL
Sjogren's anti-SS-B	1.1	0.0-9.9 U/mL
Anti-Jo-1 antibody	<1.0 (negative)	Negative: <10.0 U/mL, positive: ≥10.0 U/mL
Aquaporin 4 antibody	1.6	0.0-2.9 U/mL

Contrast-enhanced computed tomography (CT) was performed to evaluate for pulmonary thromboembolism, with no evidence of pulmonary thromboembolism or thymoma observed. Echocardiography showed normal heart function, with an ejection fraction of 55% using the Pombo method.

Her level of consciousness worsened shortly after entering the inpatient ward, with PaCO_2_ retention increasing to a level of 87.3 mmHg. Her respiratory status and level of consciousness improved approximately 30 minutes after receiving non-invasive positive pressure ventilation (NPPV) with the following settings: inspiratory positive airway pressure set at 10 cmH_2_O, expiratory positive airway pressure set at 4 cmH_2_O, respiratory rate at 14 breaths/min, fraction of inspired oxygen at 0.21. During the initial days of admission, she required continuous NPPV because her respiratory status worsened shortly after attempts were made to discontinue NPPV.

We considered the possibility of medication side effects or neuromuscular diseases in our diagnosis, as there was no medical history or imaging evidence indicating a respiratory condition. Other than dyspnea, there were no symptoms suggestive of cholinergic crisis; however, we considered cholinergic crisis, caused by a cholinesterase inhibitor administered for dementia a month before the visit, as a differential diagnosis. Memantine hydrochloride and donepezil hydrochloride were discontinued, but her respiratory status did not improve. Neurological findings showed mild bilateral iliopsoas muscle weakness (manual muscle testing {MMT} score decreased to 4), but It was challenging for us to determine whether the decrease in MMT was due to aging or an underlying medical condition. Her limb muscle strength, reflexes, and sensation were intact. No ptosis, diplopia, or bulbar symptoms were observed. Head CT and magnetic resonance imaging for the evaluation of intracranial disease showed no obvious abnormalities. A lumbar puncture revealed no evidence of Guillain Barre syndrome. While her hypokalemia corrected to within the reference range, respiratory distress did not improve. Nerve conduction and repetitive stimulation tests showed no abnormal findings. On day 13 of admission, she tested positive for anti-MuSK antibody, leading to a diagnosis of MG. Negative findings were observed for anti-nuclear antibody, anti-Sjogren’s syndrome-B antibody, anti-Jo-1 antibody, and anti-aquaporin 4 antibody. The anti-Sjogren’s syndrome-A antibody level was 21.2 U/mL (reference range: 0.0-9.9 U/mL). After her respiratory status improved with intravenous immunoglobulin and tacrolimus for MG, NPPV was discontinued. She was able to wean off NPPV on day 25 of admission. Prednisolone was introduced in addition to acute immunotherapy and pyridostigmine was also initiated as symptomatic therapy. With these acute treatments, the activities of daily living improved to the extent that the patient was able to walk with supervision and take oral intake. Although she had no complications of note during her hospitalization, as progressive disuse and dementia made it difficult for her to be discharged home, she was transferred to a hospital for rehabilitation on day 77 of admission.

## Discussion

MG is the most common neuromuscular disorder, characterized by fluctuating muscle weakness that worsens with use and improves with rest [1]. Its symptoms vary depending on the age of onset and the antibodies involved [1].

MG can present with the following signs and symptoms: ptosis in 71.9% of cases, diplopia in 47.3%, weakness of facial muscles in 5.3%, bulbar symptoms in 14.9%, weakness of extremities in 23.1%, and dyspnea in 2.3% [6]. Ocular symptoms are the most common initial presenting complaint. However, the patient did not exhibit typical ocular symptoms. Although MuSK-positive MG tends to manifest atypical symptoms, there are three distinct clinical patterns of MuSK-positive MG as follows: oculopharyngeal weakness, with occasional profound tongue and facial atrophy; neck, shoulder, and respiratory weakness without ocular involvement; and a phenotype indistinguishable from AChR-MG [4]. Our patient exhibited a type of MuSK-positive MG characterized by neck, shoulder, and respiratory weakness without ocular involvement.

Respiratory muscle weakness can be a life-threatening symptom as it may lead to pneumonia and type 2 respiratory failure [8]. Myasthenic crisis, defined as any exacerbation of MG necessitating mechanical ventilation, occurred in 7.7% of cases within one year after the onset of initial symptoms. The overall frequency of crisis was 13.3% throughout the whole period [6]. Previous studies have reported that in 2% of MuSK-positive MG cases, the initial symptom was respiratory failure, and MuSK-positive MG patients can present with selective respiratory muscle weakness without the typical ocular and limb symptoms [4,9]. Our elderly female patient also exhibited type 2 respiratory failure at the initial onset, but there were no other typical symptoms except for iliopsoas weakness, the clinical significance of which was challenging to determine due to complicating factors.

Given that MuSK-positive MG is more common in younger individuals, especially females under 40 years of age, and rarely occurs after 70 years of age, our case is considered even rarer [3]. In the elderly, physical findings are difficult to recognize due to age-related muscle weakness and the effects of medications, such as cholinesterase inhibitors for dementia. Consequently, without MG being considered as a differential diagnosis, the diagnosis can be easily delayed. Previous studies have reported an increasing incidence of late-onset and elderly-onset MG [5,6]. So it is important to be aware that we may encounter elderly patients who are initially noted to have MG.

In terms of treatment and prognosis, control agents such as pyridostigmine have been found to be less effective in managing MuSK-positive MG compared to other MG subgroups [1]. The primary treatment approach for MuSK-positive MG treatment is immunosuppression [2]. Initially, this subtype was associated with more severe symptoms, including myasthenic crisis, and a worse prognosis than other subtypes. However, recent reports suggest that MuSK-positive MG can respond well to treatment suggesting that it might not always imply a poor prognostic factor [10]. Furthermore, patients with late-onset MG, including the elderly onset, tend to have better outcomes in terms of drug requirements, drug responsiveness, and the time required to wean off ventilation when experiencing myasthenic crisis. In our case, the patient was able to wean off NPPV with acute treatment and improved the activities of daily living. It is important to be fully aware of late-onset MG and atypical cases that exhibit type 2 respiratory failure, as proper diagnosis and treatment can improve prognosis.

## Conclusions

In conclusion, this case underscores the atypical presentation of MuSK-positive MG in an elderly patient, initially manifested as type 2 respiratory failure. Some patients with MG do not exhibit the typical ocular or limb symptoms and, instead, develop respiratory failure. In the elderly, factors, such as comorbidities, medications, and age-related muscle weakness, can delay the diagnosis of MG. In elderly patients with type 2 respiratory failure, MG should be suspected even in the absence of typical symptoms.
